# Glycemic Control and Radiographic Manifestations of Tuberculosis in Diabetic Patients

**DOI:** 10.1371/journal.pone.0093397

**Published:** 2014-04-03

**Authors:** Chen-Yuan Chiang, Jen-Jyh Lee, Shun-Tien Chien, Donald A. Enarson, You-Cheng Chang, Yi-Ting Chen, Ting-Yu Hu, Chih-Bin Lin, Chi-Won Suk, Jui-Ming Tao, Kuan-Jen Bai

**Affiliations:** 1 International Union Against Tuberculosis and Lung Disease, Paris, France; 2 Division of Pulmonary Medicine, Department of Internal Medicine, Wan Fang Hospital, Taipei Medical University, Taipei, Taiwan; 3 Department of Internal Medicine, School of Medicine, College of Medicine, Taipei Medical University, Taipei, Taiwan; 4 Department of Internal Medicine, Tzu Chi General Hospital and Tzu Chi University, Hualien, Taiwan; 5 Chest Hospital, Department of Health and Welfare, Tainan County, Taiwan; 6 School of Respiratory Therapy, College of Medicine, Taipei Medical University, Taipei, Taiwan; Cambridge University, United Kingdom

## Abstract

**Background:**

Radiographic manifestations of pulmonary tuberculosis (TB) in patients with diabetes mellitus (DM) have previously been reported, with inconsistent results. We conducted a study to investigate whether glycemic control has an impact on radiographic manifestations of pulmonary TB.

**Methods:**

Consecutive patients with culture-positive pulmonary TB who had DM in three tertiary care hospitals from 2005–2010 were selected for review and compared with a similar number without DM. Glycemic control was assessed by glycated haemoglobin A1C (HbA1C). A pre-treatment chest radiograph was read independently by two qualified pulmonologists blinded to patients’ diabetic status. Films with any discordant reading were read by a third reader.

**Results:**

1209 culture positive pulmonary TB patients (581 with DM and 628 without DM) were enrolled. Compared with those without DM, TB patients with DM were significantly more likely to have opacity over lower lung fields, extensive parenchymal lesions, any cavity, multiple cavities and large cavities (>3 cm). The relative risk of lower lung field opacities was 0.80 (95% CI 0.46–1.42) for those with DM with A1C<7%, 2.32 (95% CI 1.36 - 3.98) for A1C 7%–9%, and 1.62 (95% CI 1.12–2.36) for A1C>9%; and that of any cavity over no cavity was 0.87 (95% CI 0.46–1.62) for patients with DM with A1C<7%, 1.84 (95% CI 1.20–2.84) for A1C 7%–9%, and 3.71 (95% CI 2.64–5.22) for A1C>9%, relative to patients without DM.

**Conclusions:**

Glycemic control significantly influenced radiographic manifestations of pulmonary TB in patients with DM.

## Introduction

The International Diabetes Federation has estimated that there were 382 million people living with diabetes mellitus (DM) worldwide in 2013 and the numbers will rise to 592 million by 2035 [1]. The association between DM and tuberculosis (TB) has been recently reviewed. Random effects meta-analysis of cohort studies showed that DM was associated with an increased risk of TB (relative risk 3.11, 95% CI 2.27–4.26), while risk estimated by case-control studies was heterogeneous with odds ratios ranging from 1.16 to 7.83 [2]. The rising epidemic of DM may have a significant impact on global TB control. Thus it is instructive to investigate the influence of diabetes on the manifestations of TB.

Radiographic manifestations of pulmonary TB in patients with DM have previously been reported. However, the results reported by different researchers have not been consistent. Several studies reported that TB patients with DM had an increased frequency of lower lung field lesions as compared TB patients without DM [3]–[8], but others did not [9]–[11]. Some studies reported that TB patients with DM had a higher frequency of cavitation as compared with TB patients without DM[3], [4], [6], [11]–[16], while others did not [17]–[20]. Agrawal and Chang reported that extent of disease was more advanced among TB patients with DM but Alisjahbana did not [4], [13], [19]. A major limitation of these studies has been that the number of TB patients with DM assessed was usually small and that the influence of sex and age on radiographic manifestations of pulmonary TB was not consistently addressed.

As radiographic manifestation of pulmonary TB is likely to be associated with immune status and the risk of developing TB among patients with DM is likely dependent on glycemic control [20], we hypothesized that glycemic control has an impact on radiographic manifestations of tuberculosis in patients with DM. We report the results of a study that addressed the association of DM and the influence of glycemic control on radiographic manifestations of tuberculosis.

## Materials and Methods

This study was approved by the Joint Institute Review Board of Taipei Medical University and patients’ written informed consent was waived. Patient information was anonymized and de-identified prior to analysis.

The study was conducted in three tertiary-care hospitals located in Northern, Eastern and Southern Taiwan at which the investigators work. A list of all TB patients treated at the three hospitals notified to health authorities from 2005–2010 was obtained from the national TB registry at Taiwan Center for Disease Control. Notification data and patients’ medical records maintained at hospitals were reviewed to identify all culture-positive pulmonary TB patients. Age, sex and type of TB were recorded from the notification record. Type of TB was defined as new or previously treated, according to what was recorded. A new TB patient was defined as a patient who has never been previously treated with anti-TB drugs for as long as one month. A previously treated TB patient was defined as a patient who has been previously treated with anti-TB drugs for one month or more. Their clinical records were reviewed to determine their recorded smoking habits, investigations for DM and whether or not they had a chest radiograph. Patients with DM were defined as those who 1) were treated with insulin or diabetes-specific hypoglycemic agents, 2) had been assigned an ICD-9 code related to DM during admission, 3) had been assigned an ICD-9 code related to DM 2 times or more on outpatient visits, or 4) had a history of DM. Patients who had transitory hyperglycemia at the initiation of anti-TB treatment were not included.

In order to determine the association of chest radiographic abnormalities with DM, a culture-positive pulmonary TB patient without DM who was notified to the health authority immediately prior to each culture-positive pulmonary TB patient with DM was selected for comparison. These patients were those who had never been documented to have 1) HbA1C >6.5%, or 2) fasting plasma glucose >126 mg/dl, or 2) post-prandial plasma glucose >200 mg/dl, or 3) random plasma glucose >200 mg/dl. Medical charts were reviewed for data collection by using a structured questionnaire. Glycemic control was assessed by glycated haemoglobin A1C (HbA1C) measured within 3 months of the initiation of TB treatment; diabetes patients were categorized into 3 groups: HbA1C<7%, HbA1C 7–9%, HbA1C>9%.

A pre-treatment postero-anterior chest radiograph (taken within 30 days of initiation of TB treatment) of each patient was collected by research assistants and read by two qualified pulmonologists (readers) at each hospital.

Reading of the chest radiographs focused on lung parenchymal opacity and cavitation. Recording of abnormal opacity of the lung parenchyma includes location (right upper, right lower, left upper, and left lower) and extent of disease (minimal, moderately-advanced, and far advanced). For the purpose of this study, these were defined as follows. Both right and left lung parenchyma were divided into upper and lower lung field by a horizontal line across the mid-point of a vertical line from apex to diaphragm without taking the anatomy of the lung into consideration. Extent of disease was estimated by the sum of all areas of abnormality in which a boundary of abnormal opacity could be drawn. Minimal lesions were defined as an area less than that above a horizontal line across the 2^nd^ chondrosternal conjunction of one lung; moderately-advanced lesions were defined as an area more than minimal lesions but less than one entire lung; far advanced lesions were defined as an area equivalent to or greater than one lung. Recording of cavitation included location (right upper, right lower, left upper, and left lower), number (single or multiple) and size of the largest cavity. A cavity was defined as lucency with a diameter of at least one centimeter which was generally rounded shape and could not be explained by overlapping structures (ribs, vessels or opacities). The size of the largest cavity was dichotomized into small and large by the median diameter.

The reading environment was standardized at each hospital. The default image of electronic chest radiograph was used for reading; zooming of image was discouraged. Reading was independent without discussion between readers blinded to patients’ diabetic status. Films with any discordant reading were read by a third reader, who was a senior pulmonologist at each hospital. After reading a set of 100 films, discordant films were discussed among the three readers of each hospital aiming at achieving consensus on reading. After discussion, the second set of 100 films was read independently by 2 readers and discordant films were again read by a third senior reader followed by discussion. This exercise was continued till all films of enrolled patients at each hospital were read.

Reading of chest radiographs was recorded on an electronic standard reading form using EpiData Entry 3.1 (The EpiData Association, Odense, Denmark). STATA Version 12 (StataCorp LP, College Station, Texas, USA) was used for statistical analysis. The presence of abnormal parenchymal opacity, location of opacity, extent of disease, presence of cavity, location of cavity, number of cavities and size of the largest cavity in were analyzed in relation to diabetes and glycemic control by Pearson Chi-square test. Logistic regression models were constructed for outcome variables with 2 categories and multinomial logistic regression for that with 3 categories or more and adjusted for sex, age and smoking. A p-value less than 0.05 was considered statistically significant.

## Results

A total of 797 culture-positive pulmonary TB patients with probable DM were identified and 797 culture-positive TB patients without DM were selected. Of the 797 patients with probable DM, 717 were confirmed to have DM. Of them, 1209 (79.9%) had a pretreatment chest radiograph available for assessment, 581 (81.0%) with DM and 628 (78.8%) without DM. Of the 1209 patients, 895(74.0%) were male; 1050(86.9%) were new TB patients; 535(44.3%) were ever smokers (Table 1). Sex, age, and smoking were statistically significantly associated with DM. Of the 581 patients with DM, 470 (80.9%) were diagnosed with DM prior to the diagnosis of TB; 65 (11.2%) had pre-treatment HbA1C<7%, 117 (20.1%) 7%–9%, 233 (40.1%) >9%, and 166 (28.6%) had no information of HbA1C at the initiation of anti-TB treatment.

**Table 1:** Characteristics of patients with culture-positive pulmonary tuberculosis (TB) with and without diabetes mellitus (DM) in three tertiary-referral hospitals in Taiwan, 2005–2010.

			Diabetes		
		Total	Yes	No	P-value
		N (col %)	N (col %)	N (col %)	
All		1209(100.0)	581(100.0)	628(100.0)	
Sex					0.002
	Male	895(74.0)	454(78.1)	441(70.2)	
	Female	314(26.0)	127(21.9)	187(29.8)	
Age group (years)					<0.001
	<35	132(10.9)	13(2.2)	119(19.0)	
	35–44	124(10.3)	51(8.8)	73(11.6)	
	45–54	243(20.1)	134(23.1)	109(17.4)	
	55–64	221(18.3)	156(26.9)	65(10.4)	
	65–74	208(17.2)	111(19.1)	97(15.5)	
	>75	281(23.2)	116(20.0)	165(26.3)	
Smear					<0.001
	Positive	698(57.7)	391(67.3)	307(48.9)	
	Negative	416(34.4)	148(25.5)	268(42.7)	
	Unknown	95(7.9)	42(7.2)	53(8.4)	
Type of TB					0.561
	New	1050(86.9)	508(87.4)	542(86.3)	
	Previously treated	159(13.2)	73(12.6)	86(13.7)	
Smoking					0.001
	Never	653(54.0)	285(49.1)	368(58.6)	
	Ever	535(44.3)	288(49.6)	247(39.3)	
	Unknown	21(1.7)	8(1.4)	13(2.1)	

Of the 1209 chest radiographs, 1192(98.6%) had abnormal opacity of the lung parenchyma; 1126 (93.1%) of them had opacity over upper lung fields; 862 (73.1%) had opacity over lower lung fields; 66(5.5%) had isolated lower lung field lesions without upper lung field lesions. The extent of opacity was determined; 380 (31.4%) had minimal parenchymal lesions, 572 (47.3%) moderately-advanced and 240(19.9%) far advanced. Cavitation was recorded; 490(40.5%) had cavitary lesions; 455 (37.6%) had cavities over upper lung field, and 106 (8.8%) had cavities over lower lung field; 237 (19.6%) had a cavity >3.0 cm.

Radiographic manifestations of TB differed by sex and age. Males were significantly more likely to have any opacity on lung parenchyma (male 99.3% vs female 97.6%, p = 0.019), opacity over upper lung field (male 95.8% vs female 86.9%, p<0.001), but not opacity over lower lung field (male 74.0% vs female 69.2%, p = 0.115). Females were significantly more likely to have isolated lower lung field opacity (male 3.5% vs female 10.7%), with an adjusted odds ratio 2.5 (95% CI 1.4–4.5, adjusted for age, DM and smoking). Males were significantly more likely to have far advanced parenchymal lesions (male 23.0% vs female 14.5%, p<0.001), any cavitary lesion (male 46.3% vs female 31.5%, p<0.001) and cavitary lesions over upper lung fields (male 43.2% vs female 28.7%, p<0.001) but not cavitary lesions over lower lung fields (male 10.0% vs female 6.9%, p = 0.116).

**Figure 1:**
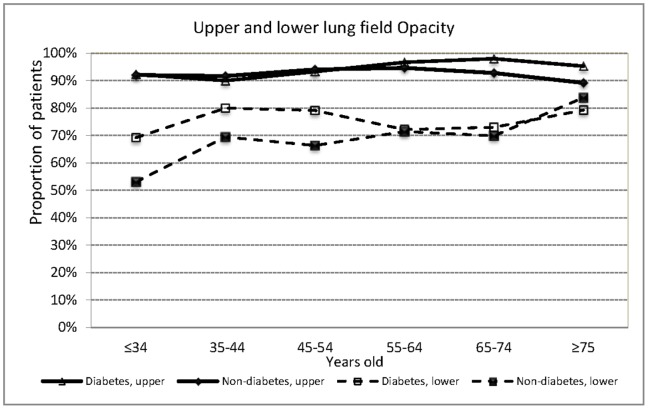
Location of radiographic opacity by age groups in culture positive pulmonary tuberculosis patients with and without diabetes mellitus (DM) in three tertiary-referral hospitals in Taiwan, 2005–2010.

**Figure 2:**
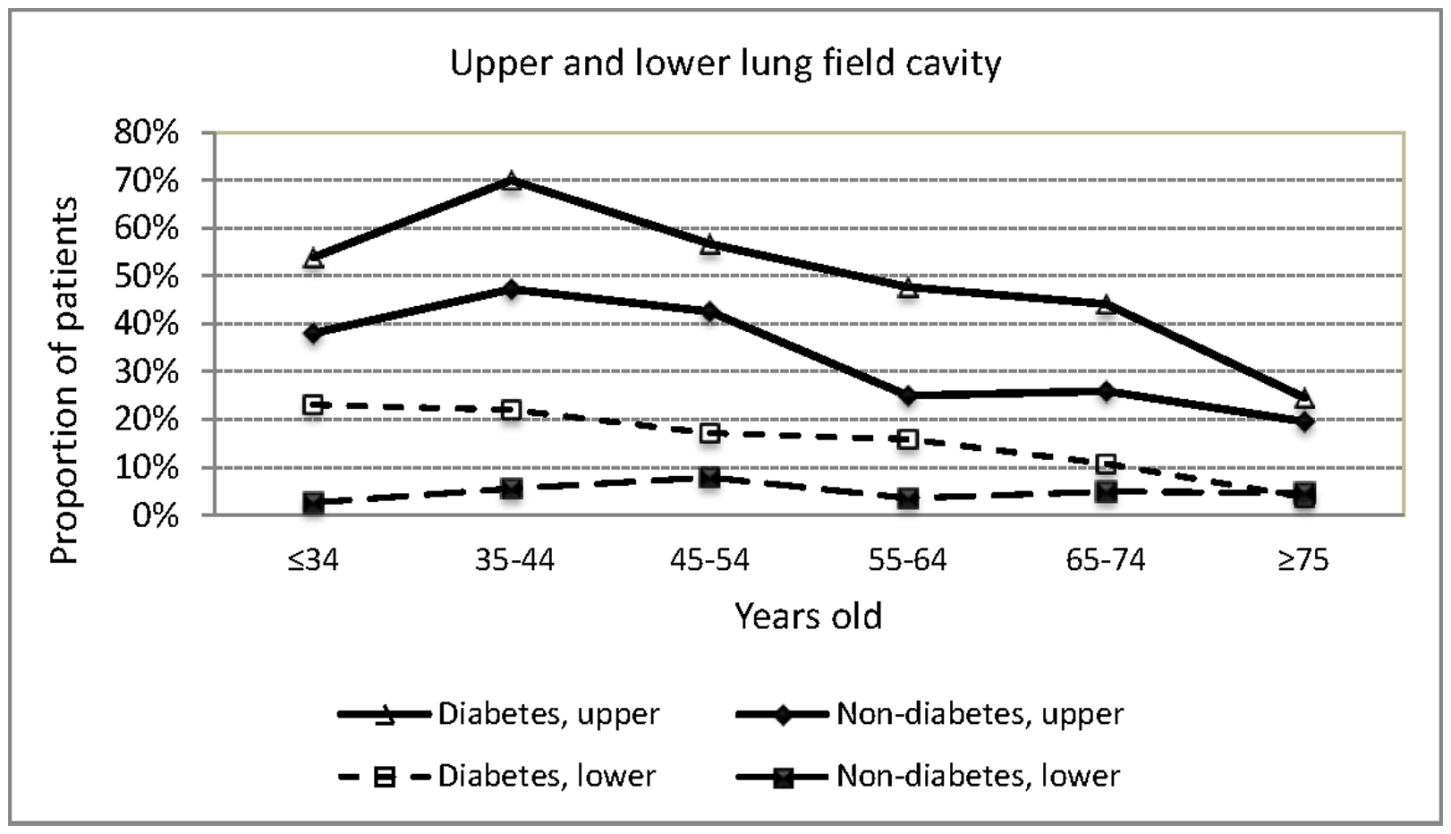
Location of radiographic cavities by age groups in culture positive pulmonary tuberculosis patients with and without diabetes mellitus (DM) in three tertiary-referral hospitals in Taiwan, 2005–2010.

The proportion of patients with upper lung field opacity did not differ by age group (p = 0.380). Those aged 65 years or older were significantly more likely to have lower lung field opacity than those <65 years old (75.3% vs 68.6%, adjusted OR 1.42, 95% CI 1.09–1.85, adjusted for sex, diabetes and smoking). In all age groups, the proportion of patients with upper lung field opacity was higher than those with lower lung field opacity. The difference between the proportion with upper and that with lower lung field opacity was particularly striking in those aged <35 years but less so in those aged 75 years or more because the proportion of patients with lower lung field opacity was highest among patients aged >75 years (80.4%) and lowest among those aged <35 years(53.1%) (p<0.001). Those aged 65 years or older were significantly less likely to have cavitary lesions than those <65 years old (27.6% vs 49.3%, p<0.001). The proportion of patients with cavitary lesions was highest among those aged 35–44 years (58.1%). In all age groups, the proportion of patients with upper lung field cavity was higher than that with lower lung field cavity. Age group was significantly associated with upper lung field cavity (p<0.001) and lower lung field cavity (p = 0.001). When stratified by DM, the proportion of patients with upper lung field opacity was higher than that with lower lung field opacity in all age groups in both those with and without DM; the same for cavitary lesion. However, due to the increased proportion of patients with lower lung field opacity among those with DM in those aged <55 years old, the association between age group and lower lung field opacities was no longer statistically significant among diabetes (p = 0.550). (figure 1) In terms of cavity, there was an increased proportion of both patients with upper lung field cavity and patients with lower lung field cavity among those with DM in those aged <65 years old (figure 2). Age group was significantly associated with upper lung field cavity in both those with (p<0.001) and without DM (p<0.001); age group was significantly associated with lower lung filed cavity in those with DM (p = 0.003) but not in those without DM (p = 0.613).

Table 2 shows the association of location and extent of radiographic opacity of lung parenchyma with DM. Compared with those without DM, TB patients with DM were significantly more likely to have opacity over lower lung fields and extensive parenchymal lesions (moderately or far advanced). Table 3 shows the association of location, number and size of cavitary lesions of lung parenchyma with DM. Compared with patients without DM, TB patients with DM were significantly more likely to have any cavity (51.6% vs 34.4%, p<0.001), cavitary lesions over upper lung fields (47.1% vs 32.8%, p<0.001) and cavitary lesions over lower lung fields (13.7% vs 4.9%, p<0.001); these associations were all statistically significant in multivariate analysis adjusted for sex, age and smoking. TB patients with DM were also significantly more likely to have multiple cavities and large cavities (>3 cm) as compared with patients without DM.

**Table 2:** Location and extent of abnormal opacities of lung parenchyma on chest radiograph in culture positive pulmonary tuberculosis patients with and without diabetes mellitus (DM) in three tertiary-referral hospitals in Taiwan, 2005–2010.

	Diabetes mellitus							
	Yes		No		Univariate		Multivariate	
	No.	Col %	No.	Col %	OR	(95% CI)	AdjOR	(95% CI)
Total	581	100.0	628	100.0				
Any opacity	575	99.0	617	98.3	1.71	(0.63–4.65)	1.52	(0.53–4.35)
**Location**								
Upper field	550	94.7	576	91.7	1.60	(1.01–2.54)	1.38	(0.84–2.25)
Right	448	77.1	491	78.2	0.94	(0.72–1.23)	0.81	(0.60–1.09)
Left	402	69.2	419	66.7	1.12	(0.88–1.45)	1.01	(0.78–1.32)
Lower field	438	75.4	424	67.5	1.47	(1.15–1.90)	1.37	(1.04–1.81)
Right	330	56.8	348	55.4	1.06	(0.84–1.33)	0.97	(0.76–1.25)
Left	337	58.0	313	49.8	1.39	(1.11–1.74)	1.32	(1.03–1.69)
Isolated lower	25	4.3	41	6.5	0.64	(0.39–1.07)	0.76	(0.44–1.30)
Right field	483	83.1	541	86.2	0.79	(0.58–1.08)	0.71	(0.50–1.00)
Left field	457	78.7	472	75.2	1.22	(0.93–1.59)	1.12	(0.83–1.50)
Bilateral field	365	62.8	396	63.1	0.99	(0.78–1.25)	0.88	(0.68–1.13)
**Extent**								
Minimal	157	27.0	223	35.5	Ref		Ref	
Moderately-advanced	297	51.1	275	43.8	1.53	(1.18–1.99)	1.53	(1.15–2.03)
Far-advanced	121	20.8	119	19.0	1.44	(1.04–2.00)	1.31	(0.92–1.87)

Note: No, number; OR, odds ratio; CI, confidence interval; AdjOR, adjusted odds ratio, adjusted for sex, age group, and smoking.

**Table 3:** Location and size of cavitary lesions of lung parenchyma on chest radiograph in culture positive pulmonary tuberculosis patients with and without diabetes mellitus (DM) in three tertiary-referral hospitals in Taiwan, 2005–2010.

	Diabetes mellitus							
	Yes		No		Univariate		Multivariate	
	No	Col %	No	Col %	OR	(95% CI)	AdjOR	(95% CI)
Total	581	100.0	628	100.0	–	–	–	–
Any Cavity	290	49.9	200	31.9	2.13	(1.69–2.69)	2.27	(1.74–2.96)
Location (field)								
Upper	265	45.6	190	30.3	1.93	(1.52–2.45)	2.04	(1.56–2.67)
Right	158	27.2	119	19.0	1.60	(1.22–2.09)	1.55	(1.15–2.10)
Left	150	25.8	125	19.9	1.40	(1.06–1.83)	1.36	(1.01–1.83)
Lower	77	13.3	29	4.6	3.16	(2.03–4.92)	2.83	(1.76–4.53)
Right	43	7.4	12	1.9	4.10	(2.14–7.86)	3.84	(1.90–7.75)
Left	42	7.2	21	3.3	2.25	(1.32–3.85)	2.09	(1.18–3.70)
Isolated lower	14	2.4	5	0.8	3.08	(1.10–8.60)	2.50	(0.84–7.45)
Right	175	30.1	126	20.1	1.72	(1.32–2.24)	1.67	(1.25–2.24)
Left	169	29.1	132	21.0	1.54	(1.19–2.00)	1.52	(1.13–2.03)
Bilateral	54	9.3	58	9.2	1.01	(0.68–1.49)	0.87	(0.57–1.32)
Number of cavities*								
Single	121	20.8	81	12.9	2.20	(1.60–3.02)	2.54	(1.79–3.64)
Multiple	169	29.1	119	19.0	2.09	(1.58–2.76)	2.10	(1.53–2.87)
Size of cavity*								
<3 cm	146	25.1	107	17.0	2.00	(1.50–2.68)	2.22	(1.61–3.07)
≥3 cm	144	24.8	93	14.8	2.28	(1.69–3.08)	2.32	(1.65–3.27)

Note: No, number; OR, odds ratio; CI, confidence interval; AdjOR, adjusted odds ratio, adjusted for sex, age group, and smoking; large cavity, diameter 3 cm or bigger.

*No cavity as base for comparison.

Glycemic control significantly influenced radiographic manifestations of pulmonary TB. The proportion of patients with lower lung field opacities was 67.5% in those without DM, 67.7% in those with DM with A1C<7%, 83.8% with A1C 7%–9%, and 76.8% with A1C>9%. From multivariate logistic regression analysis adjusted for sex, age and smoking, the relative risk of lower lung field opacities was 0.80 (95% CI 0.46–1.42) for those with DM with A1C<7%, 2.32 (95% CI 1.36 - 3.98) for A1C 7%–9%, and 1.62 (95% CI 1.12–2.36) for A1C>9%, compared with patients without DM. The proportion of patients with extensive (moderately advanced or far advanced) pulmonary parenchymal lesions was 62.7% among those without DM, 58.5% in those with DM with A1C<7%, 74.4% with A1C 7%–9%, and 76.8% with A1C>9% (p<0.001). From multivariate logistic regression analysis adjusted for sex, age and smoking, the relative risk of extensive parenchymal lesions was 0.74 (95% CI 0.43–1.27) for those with DM with A1C<7%, 1.66 (95% CI 1.04–2.63) for A1C 7%–9%, and 2.05 (95% CI 1.41–2.98) for A1C>9%, relative to those without DM.

**Figure 3:**
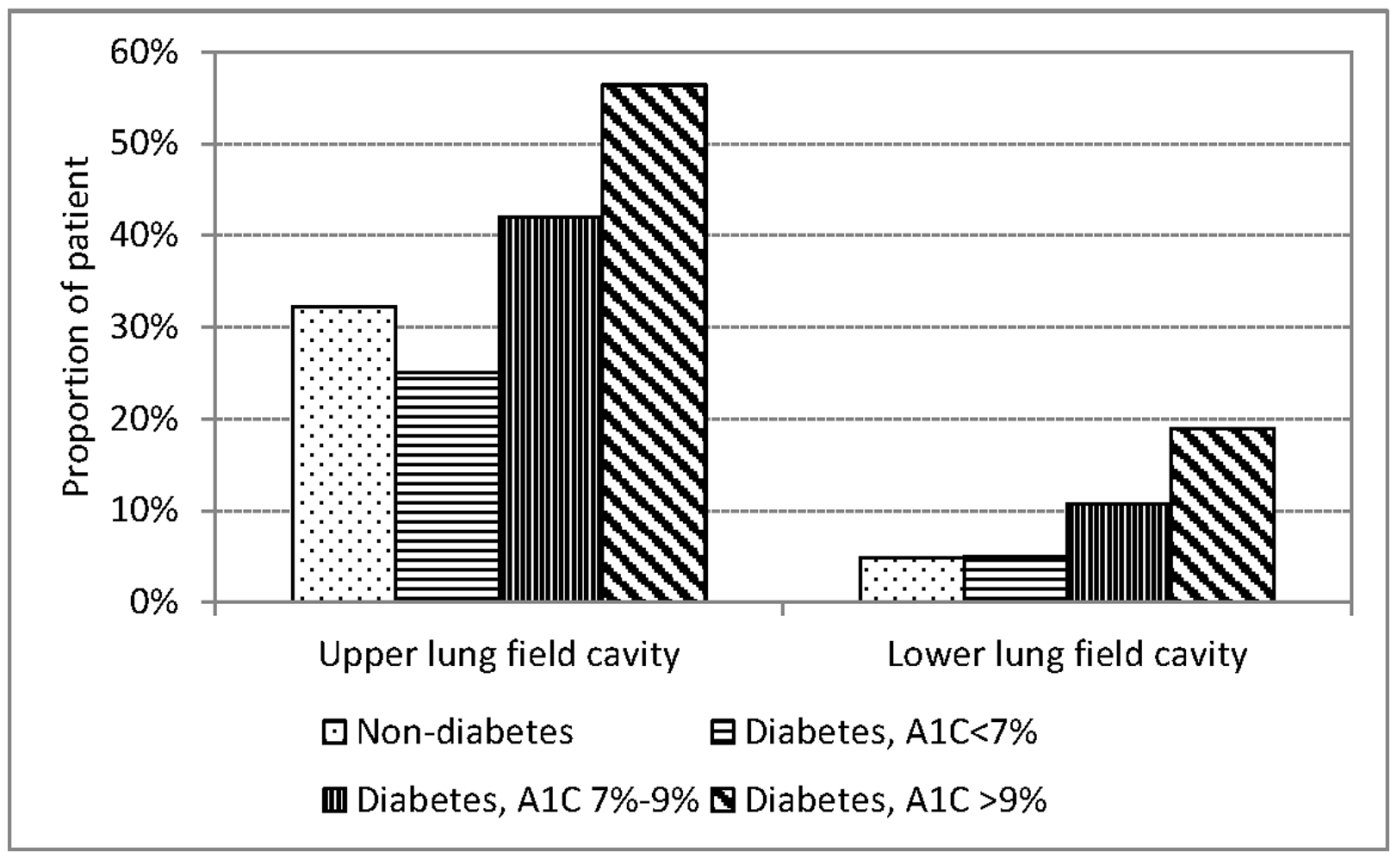
Location of cavitary lesions in culture positive pulmonary tuberculosis patients with and without diabetes mellitus by hemoglobin A1C level in three tertiary-referral hospitals in Taiwan, 2005–2010.

**Figure 4:**
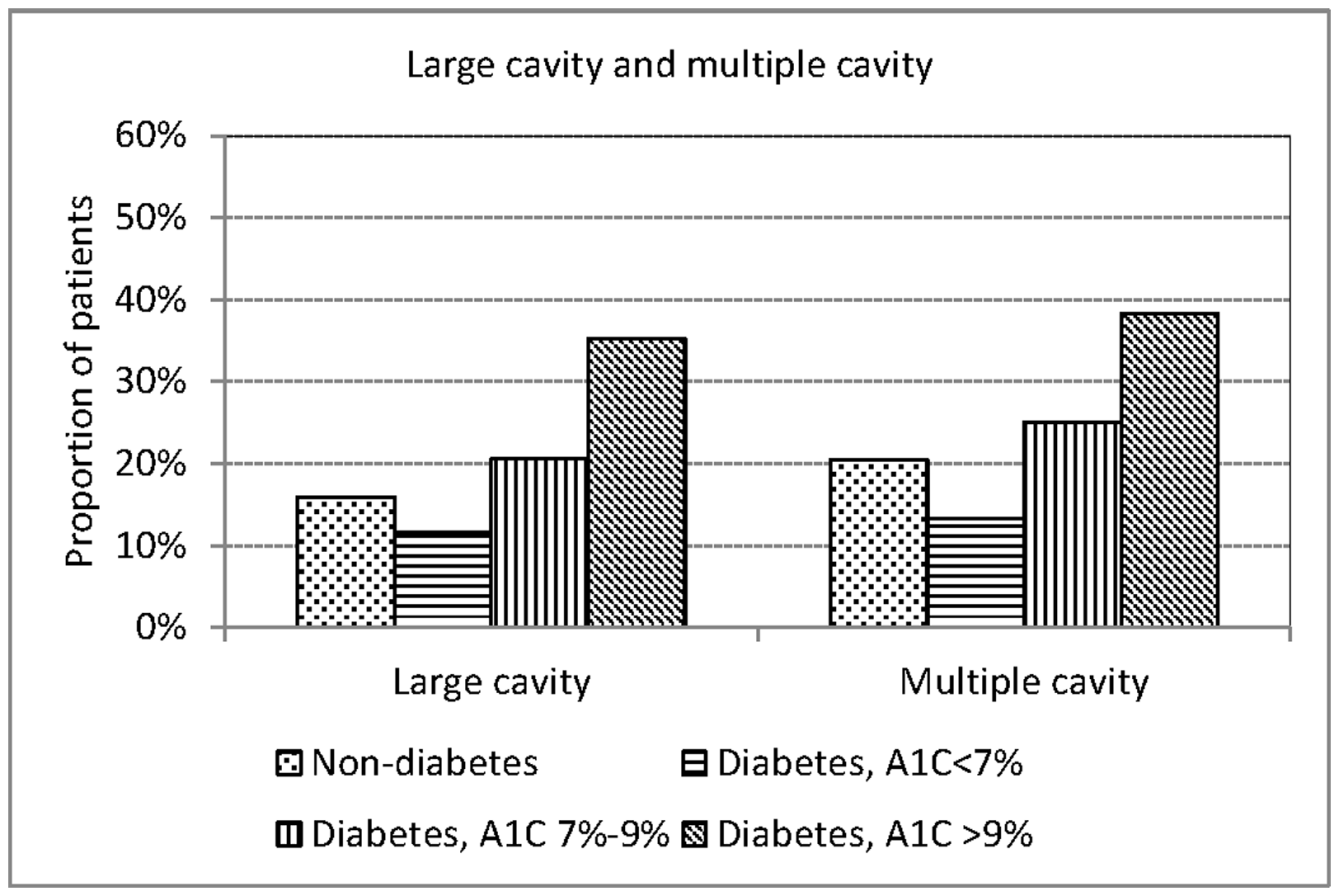
Presence of large or multiple cavitary lesions in culture positive pulmonary tuberculosis patients with and without diabetes mellitus by hemoglobin A1C level in three tertiary-referral hospitals in Taiwan, 2005–2010.

The proportions of patients with DM with any cavity, cavitary lesions over upper lung field, cavitary lesions over lower lung field, large cavity (>3 cm) and multiple cavities were all highest among patients with A1C>9%, followed by A1C 7%–9%, and lowest among A1C<7% (figure 3 and figure 4). Table 4 shows that the association between glycemic control and any cavitary lesion, cavitary lesions over upper lung field, cavitary lesions over lower lung field, multiple cavities and large cavity were all statistically significant in multinomial logistic regression analysis adjusted for sex, age and smoking. The relative risk of any cavity over no cavity was 0.87 (95% CI 0.46–1.62) for patients with DM with A1C<7%, 1.84 (95% CI 1.20–2.84) for A1C 7%–9%, and 3.71 (95% CI 2.64–5.22) for A1C>9%, relative to patients without DM.

**Table 4:** Multinomial logistic regression models assessing glycemic control and radiographic manifestations of any cavity, cavities on upper lung fields, cavities on lower lung fields, number of cavity, and large cavity in culture positive pulmonary tuberculosis patients with and without diabetes mellitus (DM) in three tertiary-referral hospitals in Taiwan, 2005–2010.

	A1C<7%		A1C 7%–9%		A1C>9%	
	AdjRRR	(95% CI)	AdjRRR	(95% CI)	AdjRRR	(95% CI)
Any Cavity	0.79	(0.42–1.49)	2.00	(1.30–3.09)	3.59	(2.53–5.11)
Location (field)						
Upper	0.89	(0.46–1.62)	1.86	(1.20–2.88)	2.71	(1.92–3.83)
Lower	1.02	0.30–3.51)	2.28	(1.10–4.71)	4.47	(2.62–7.62)
Number of cavities						
Single	0.97	(0.41–2.29)	2.46	(1.40–4.32)	3.97	(2.53–6.25)
Multiple	0.68	0.30–1.53)	1.71	(1.02–2.88)	3.37	(2.26–5.03)
Size of cavity						
Small	0.80	(0.36–1.77)	2.20	(1.32–3.67)	3.34	(2.19–5.08)
Large	0.79	(0.34–1.88)	1.77	(1.01–3.12)	3.87	(2.54–5.90)

Note: Non-diabetic pulmonary TB patients as the base for comparison; A1C, glycated hemoglobin; adjRRR, relative risk ratio adjusted for sex, age group and smoking; CI, confidence interval; large cavity, diameter > 3 cm.

Radiographic manifestations of TB were not significantly associated with type of TB patients (new vs previously treated) in terms of opacity over upper lung field (p = 0.098), isolated lower lung field opacity (p = 0.315), extent of disease (p = 0.290), cavitary lesions over upper lung fields (p = 0.465), cavitary lesions over lower lung fields (p = 0.128), number of cavity (p = 0.107) and large cavities (p = 0.598). However, as compared with new TB patients, previously treated TB patients were significantly more likely to have opacity over lower lung field (new 70.0% vs previously treated 79.9%, p = 0.010). Restricting the analysis on location and extent of abnormal opacities and location and size of cavitary lesions of lung parenchyma in relation to DM and glycemic control in new TB patients only did not disclose any finding (data not shown) significantly different from that including both new and previously treated TB patients.

## Discussion

Several studies have reported an association between diabetes and radiographic manifestations of pulmonary TB. Most studies had a relatively small sample size. Consequently, findings of radiographic manifestations of pulmonary TB were substantially influenced by random variations, and the influence of age and sex on radiographic manifestations of pulmonary TB were not taken into account. Our study enrolled 581 culture positive pulmonary TB patients with diabetes and compared them to a similar number without diabetes. The relatively large sample size provided better power in analyzing the influence of sex, age, and diabetes on the radiographic manifestations of pulmonary TB. To our knowledge, this is the second study on the association between glycemic control and radiographic manifestations of pulmonary TB. While we were collecting data for this study, Park et al published a paper on the effect of diabetic control on the clinical features of pulmonary tuberculosis but their sample size was also relatively small (124 diabetic patients in whom 60% had HbA1C ≥7.0, 20% <7.0, and 20% had no information on HbA1C level) [21].

Our findings confirmed previous reports that age and sex were associated with radiographic manifestation of pulmonary TB [22]. Male sex is significantly more likely to be associated with far advanced parenchymal and cavitary lesions as compared with females. The elderly are more likely to have lower lung field lesions and less likely to have cavities as compared with younger patients [23]. These findings need to be taken into account in investigating the association between diabetes and radiographic manifestation of pulmonary TB.

Isolated lower lung field TB without upper lung field involvement deserves attention as diagnosis of lower lung field TB can be difficult. Aktogu reported that 6.2% of pulmonary TB patients had isolated lower lung field TB and the proportion of patients with isolated lower lung field TB was higher among females (11.8% of females vs 4.4% of males, p<0.005) and diabetes (11% of diabetic patients vs 5.3% non-diabetic patients) [5]. Chang reported that 5.1% of pulmonary TB patients had isolated lower lung field TB and the proportion of patients with isolated lower lung field TB was 16.3% among females and 3.1% among males (p<0.005) [24]. That a higher proportion of diabetic patients had isolated lower lung field TB than non-diabetes has also been reported by Pérez-Guzmán (19% among diabetes vs 7% among non-diabetes), Umut (8.1% among diabetes vs 2.7% among non-diabetes) and Marais (29% among diabetes vs 4.5% among non-diabetes) [3], [8], [14]. Our study confirmed that isolated lower lung field TB is more frequent among female than male but not more frequent among diabetes than non-diabetes.

Our study confirms previous reports that diabetic TB patients have an increased frequency of lower lung field lesions as compared with non-diabetic TB patients [3], [5], [7], [8]. The increased frequency of lower lung field involvement was mainly among the younger patients and was related to glycemic control. Diabetes with A1C<7% did not have an increased frequency of lower lung field involvement. Perez-Guzman reported that the proportion of patients with tuberculosis with lower lung field involvement progressively increased with age and proposed that age-induced changes in increased alveolar ventilation and reduced perfusion favor multiplication of *Mycobacterium tuberculosis* in lower lung zones [25]. As lower lung field involvement was common in all ages in diabetes, Perez-Guzman further suggested that diabetes and aging predispose to similar radiologic changes in patients with tuberculosis. Our data concur with findings of Perez-Guzman but we found that diabetes-related radiographic change in lower lung field opacities occurred mainly among the younger patients and was driven by glycemic control. It is possible that tight glycemic control might be able to reduce diabetes-related radiographic change in lower lung field.

In terms of cavity, Perez-Guzman reported that in patients with tuberculosis alone, cavitation became less common with age, whereas the frequency of cavitation remained high in diabetics of all ages [25]. Our data did not concur with Perez-Guzman’s observations. We observed that the proportion of patients with cavitary lesions was highest among those aged 35–44 years and decreased progressively with age. Diabetes did not obscure but aggravated the differential risks of cavitary lesions between the elderly and younger patients. Diabetes increased the risk of cavitary lesions, especially among younger patients, likely through a mechanism that is different from the one causing increased lower lung field involvement, and the risk of cavity among diabetes patients is driven by glycemic control. Park also reported that diabetic patients with poor glycemic control had an increased frequency of cavity but not diabetic patients with proper glycemic control [21]. The increased frequency of pulmonary cavitary lesions in diabetic patients with poor glycemic control is probably related to reduced expression of Th1-related cytokines. [13], [26]–[28]. It is possible that proper glycemic control will not only reduce the risk of tuberculosis among diabetes patients but also attenuates the risk of cavitary lesions of pulmonary TB in diabetic patients.
